# Colonic Stricture Secondary to Recurrent Ischemic Colitis

**DOI:** 10.7759/cureus.15478

**Published:** 2021-06-06

**Authors:** Faisal Mehmood, Amina Khalid, Sultan Mahmood

**Affiliations:** 1 Hospital Medicine, Montefiore Medical Center/Albert Einstein College of Medicine, Bronx, USA; 2 Internal Medicine, North Central Bronx Hospital, Bronx, USA; 3 Gastroenterology, University at Buffalo, Buffalo, USA

**Keywords:** abdominal pain, hematochezia, anticoagulation, colon ischemia, stricture

## Abstract

Colonic ischemia is the most common form of gastrointestinal ischemia, which frequently affects the elderly population. The diagnosis and treatment can be challenging since it is prevalent in patients who are debilitated and have multiple comorbidities. However, most cases remain undetected until further complications emerge. Some of these patients will develop prolonged complications like chronic ischemic colitis or stricture requiring surgical intervention. Here we present a case of a colonic stricture secondary to recurrent ischemic colitis in an elderly female patient with multiple medical problems.

## Introduction

The incidence of colonic ischemia has increased over time and is estimated at 16 cases per 100,000 person-years [1]. It is more prevalent in women. It presents with lower abdominal pain, hematochezia, or bloody diarrhea. However, symptoms can be non-specific and can mimic other gastrointestinal disorders. Clinical presentation can vary from mild and reversible disease to irreversible injury [2]. Clinicians should have a high degree of clinical suspicion for early diagnosis and management of colonic ischemia. Approximately 15% of patients develop acute complications including gangrene which can be life-threatening. However, some of these patients can develop long-term complications, such as chronic ischemic colitis or colonic stricture. Since disease occurs in patients with multiple comorbidities, management becomes challenging involving multi-disciplinary teams. Here we present a case of colonic stricture from recurrent episodes of colonic ischemia in an elderly female. This case was presented as poster in 2018 at ACG annual scientific meeting on October 8, 2018.

## Case presentation

An 86-year-old woman was admitted for two days history of hematochezia and intermittent abdominal pain. Her past medical history included type 2 diabetes mellitus, hypertension, heart failure with reduced ejection fraction, coronary artery disease requiring angioplasty with stenting, prosthetic mitral valve ring, and atrial fibrillation. She was taking anticoagulation therapy but was non-adherent with the regimen. She was a non-smoker and had no history of recent antibiotic or nonsteroidal anti-inflammatory drug (NSAID) use. She denied weight loss, changes in bowel habits, and family history of colon cancer. She had a previous admission at another hospital with similar complaints approximately six weeks before her current hospitalization. It was reported that a computerized tomography (CT) angiogram of the abdomen from that hospitalization was suspicious for ischemia of hepatic flexure. Images were not made available. Colonoscopy was deferred at that time as she had acute decompensated heart failure in addition to hematochezia.

On physical examination, she was afebrile and had epigastric abdominal tenderness and rectal examination with brown stool, and the guaiac test was positive.

**Figure 1:**
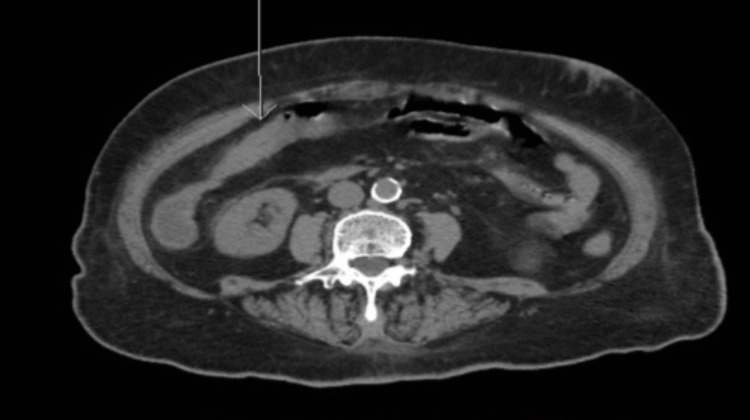
CT angiogram of abdomen demonstrating a focus of thickened and friable intestinal wall in the proximal transverse colon with minimal fatty stranding, indicating ischemia or inflammation.

**Figure 2:**
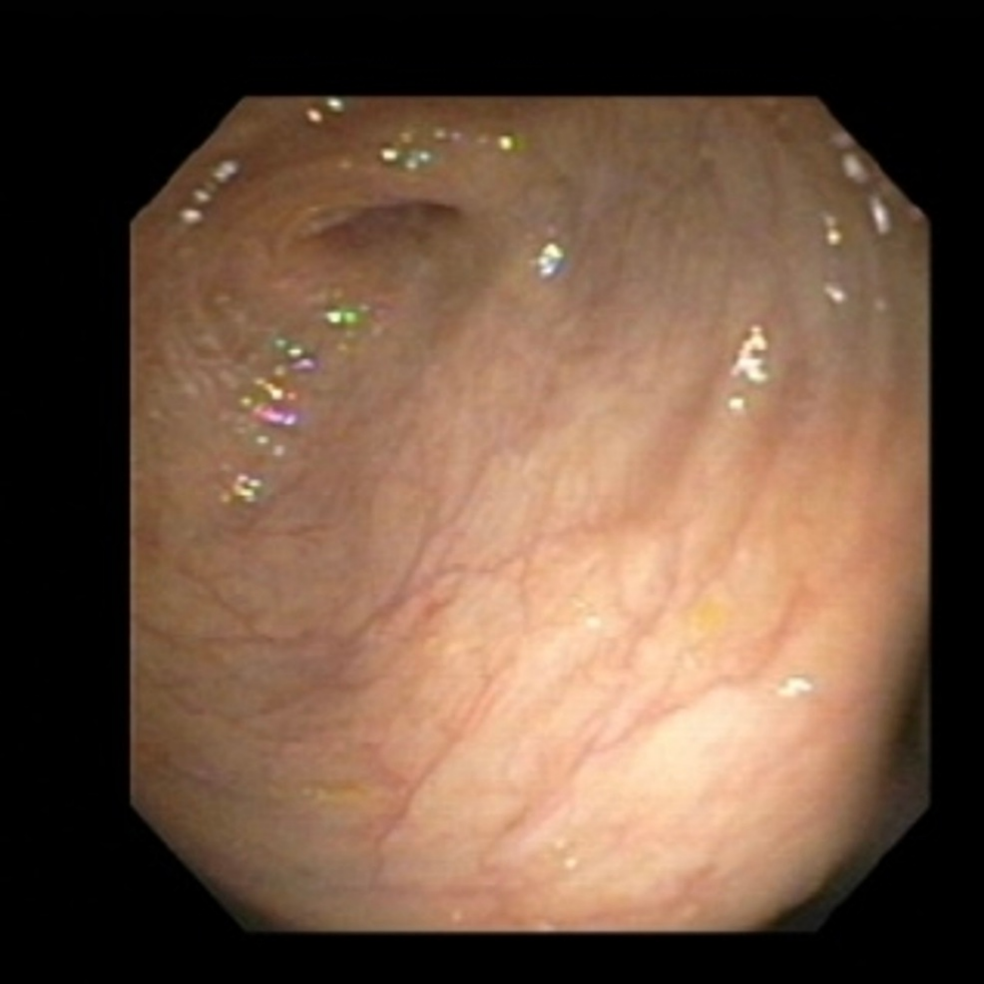
Colonoscopy showing moderate to severe colonic stenosis at the hepatic flexure.

**Figure 3:**
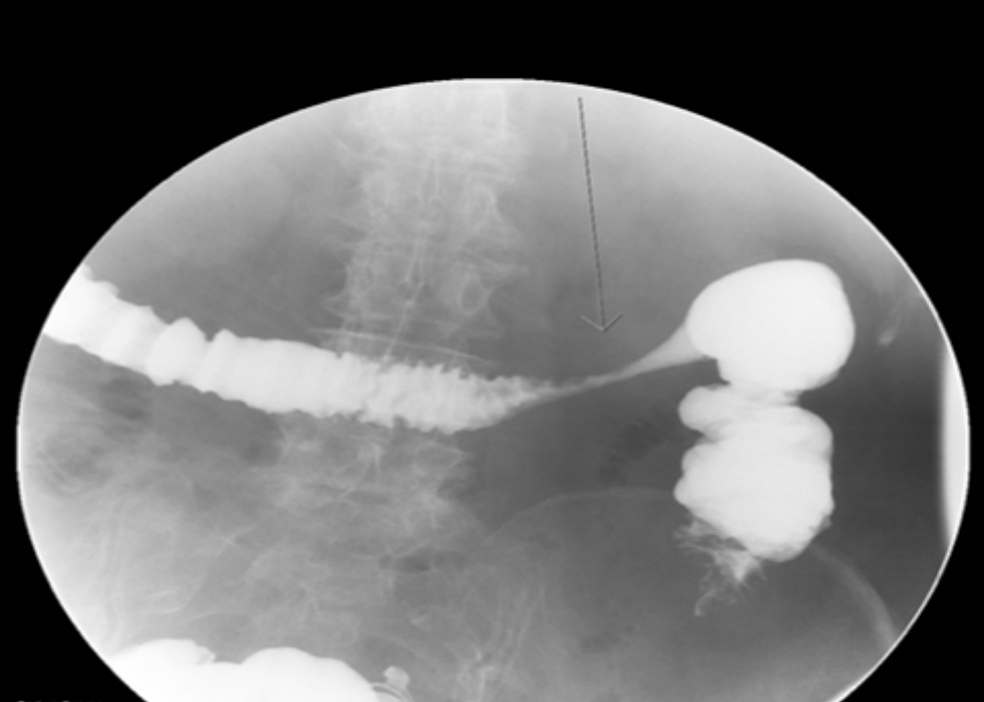
Barium enema showing a stricture demonstrating 4.4-cm-long segment of narrowing at the proximal transverse colon.

Laboratory investigations revealed new-onset anemia with a hemoglobin of 6.7 g/L from a baseline of 9-10 g/L and a subtherapeutic international normalized ratio (INR) of 1.1. Other blood test results were normal including WBCs, kidney and liver functions. CT angiogram of the abdomen showed patent celiac axis and mesenteric arteries, but a focus of thickened and friable intestinal wall in the proximal transverse colon with minimal fatty stranding, indicating ischemia or inflammation (Figure 1). GI was consulted and a colonoscopy was performed which showed moderate to severe colonic stenosis at the hepatic flexure and scope could not be passed beyond hepatic flexure (Figure 2). Biopsy was deferred due to risk of recurrent bleeding and underlying comorbidities. To further delineate characteristics of the stricture, a barium enema was performed, which revealed a 4.4-cm long segment of narrowing at the proximal transverse colon (Figure 3). This stricture formation was attributed to recurrent episodes of colon ischemia involving the same anatomical site. Etiology of ischemia was thought to be multifactorial including hypoperfusion from decompensated heart failure, atrial fibrillation, and polypharmacy.

An echocardiogram was done to look for the cardiac source of embolus which revealed moderately dilated left atrium and ejection fraction was 45%. Carcinoembryonic antigen (CEA) level was 1.6 ng/ml. General surgery was consulted and it was recommended that the patient should undergo a right-sided hemicolectomy to address the stricture; however, the patient refused the surgery. During the hospitalization, the patient received multiple blood transfusions and the hematochezia resolved spontaneously. Anticoagulation was restarted with no further bleeding. She had a family meeting to discuss the goals of care. Patient and her family deferred any further surgical or endoscopic intervention. She was discharged on medical management and was doing well on one-month post-discharge follow-up.

## Discussion

Intestinal ischemia more commonly affects the colon since it has less blood flow and the microvascular plexus in the thick wall of the colon is less developed as compared with the small intestine [3]. The pathophysiology involves an acute sudden reduction in blood flow disrupting the cellular metabolism [4]. The main mechanism of colon ischemia includes non-occlusive ischemia from hypoperfusion of the mesenteric vasculature, acute thromboembolic phenomenon in mesenteric vessels, or mesenteric vein thrombosis. Prolonged severe ischemia can lead to transmural infarction within few hours [3]. Colonic ischemia most commonly affects the "watershed" areas of the colon such as splenic flexure and rectosigmoid junction, which have limited collateral circulation of blood and are at risk for ischemia particularly related to hypoperfusion [5-7].

Post-ischemic strictures are extremely rare sequelae of ischemic bowel disease [8-9]. It is difficult to make a diagnosis of post-ischemic stricture formation in the absence of a clear history of ischemic bowel disease [10]. Diagnosis is challenging because there is an overlap of clinical symptoms and histopathological findings with other bowel diseases such as Crohn's disease, NSAID-induced enteropathy, and cryptogenic multifocal ulcerous stenosing enteritis [11]. Patient’s medication history, duration of clinical symptoms, fecal calprotectin, endoscopic appearance of colonic mucosa, and histopathology findings can be useful to differentiate the etiology of stricture formation. Management depends on the etiology. Treatment includes discontinuation of any offending agent, medical treatment with steroids and anti-inflammatory agents, endoscopic surgery including balloon dilation with stent placement or needle knife therapy to open up the stricture, and bowel resection and anastomosis or strictureplasty [12-14]. These patients should be on low fiber diet and take stool softeners to prevent any obstructive symptoms.

There are few case reports of colon stricture formation from various other rare etiologies. These include iatrogenic superior mesenteric artery injury, portal vein thrombosis, NSAID-induced, colonic cryptococcosis, and amebiasis [15-18]. To the best of our knowledge, this is the first reported case of hepatic flexure stricture attributed to recurrent ischemic episodes in the same anatomical area.

## Conclusions

Our patient had ischemia involving hepatic flexure of the colon which is not a watershed area and rarely gets ischemic events. One clue to the diagnosis was a prior ischemic event involving the same area of the colon and non-inflammatory stricture suggesting chronic colon ischemia as most likely etiology. Therefore, patient education is crucial to ensure medication adherence to prevent recurrent ischemic events to prevent chronic complications.
